# Supplementary material for: Causal Relationship between Aging and Anorexia Nervosa: A White-Matter-Microstructure-Mediated Mendelian Randomization Analysis
Source: Biomedicines. 2024 Aug 16;12(8):1874. doi: 10.3390/biomedicines12081874 (PMC11351342; doi:10.3390/biomedicines12081874)
Supplement: Supplementary file 1 [file biomedicines-12-01874-s001.zip › Supplementary Figure/Figure S8/Leaveoneout plot of MR analysis for TL to ubm-b-1769.pdf]

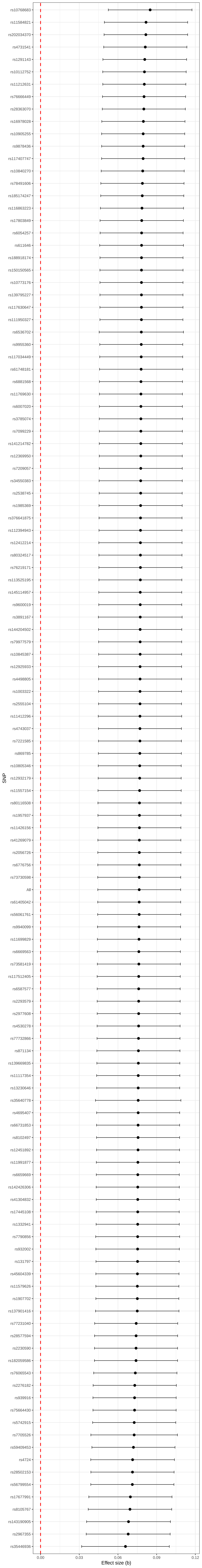
rs10768683
rs11584821
rs202034370
rs4731541
rs1291143
rs10112752
rs11212631
rs76666449
rs28363070
rs16978028
rs10905255
rs9878436
rs117407747
rs10840270
rs78491606
rs185174247
rs116863223
rs17803849
rs6054257
rs611646
rs188918174
rs150150565
rs10773176
rs139795227
rs117630647
rs111950327
rs6536702
rs9955360
rs117034449
rs61748181
rs6881568
rs11769630
rs6007020
rs3785074
rs7099229
rs141214782
rs12369950
rs7209057
rs34550383
rs2538745
rs1985369
rs376641875
rs112394943
rs12412214
rs80324517
rs76219171
rs113525195
rs145114957
rs9600019
rs3891167
rs144204502
rs79977579
rs10845387
rs12925933
rs4498805
rs1003322
rs2555104
rs11412296
rs4743037
rs7221585
rs869785
rs10805346
rs12932179
rs11557154
rs80116508
rs1957937
rs11426156
rs41269079
rs2056726
rs6776756
rs73730598
All
rs61405042
rs56061761
rs9940099
rs11699829
rs6669563
rs73581419
rs117512405
rs6587577
rs2293579
rs2977608
rs4530278
rs77732866
rs871134
rs139669835
rs11117354
rs13230646
rs35640778
rs4695407
rs66731853
rs8102497
rs12451892
rs11991877
rs6659669
rs142426306
rs41304832
rs17445108
rs1332941
rs7790856
rs932002
rs131797
rs45604339
rs11579626
rs1907702
rs137901416
rs77231040
rs28577594
rs2230590
rs182059586
rs76065543
rs2276182
rs939916
rs75664430
rs5742915
rs7705526
rs59409453
rs4724
rs28502153
rs56799554
rs17677991
rs8105767
rs143190905
rs2967355
rs35446936
SNP
0.00
0.03
0.06
0.09
0.12
Effect size (b)
